# Building and benchmarking the motivated deception corpus: Improving the quality of deceptive text through gaming

**DOI:** 10.3758/s13428-022-02028-7

**Published:** 2022-12-22

**Authors:** Dan Barsever, Mark Steyvers, Emre Neftci

**Affiliations:** Department of Cognitive Sciences, University of California, Irvine, CA USA

**Keywords:** Deception, Text, Machine learning, Neural networks, Corpus, BERT, Natural language processing, Truth, Lie

## Abstract

When one studies fake news or false reviews, the first step to take is to find a corpus of text samples to work with. However, most deceptive corpora suffer from an intrinsic problem: there is little incentive for the providers of the deception to put their best effort, which risks lowering the quality and realism of the deception. The corpus described in this project, the Motivated Deception Corpus, aims to rectify this problem by gamifying the process of deceptive text collection. By having subjects play the game Two Truths and a Lie, and by rewarding those subjects that successfully fool their peers, we collect samples in such a way that the process itself improves the quality of the text. We have amassed a large corpus of deceptive text that is strongly incentivized to be convincing, and thus more reflective of real deceptive text. We provide results from several configurations of neural network prediction models to establish machine learning benchmarks on the data. This new corpus is demonstratively more challenging to classify with the current state of the art than previous corpora.

## Introduction

One of the most basic requirements of any natural language study is the need for a quality corpus of data. Deception detection in text is no different. However, in this respect, deceptive text has an additional complication that is not present in, for example, sentiment analysis. Sentiment in text is comparatively easy for humans to identify (Vogler & Pearl, 2019) and generate, and good samples are relatively simple to gather. Quality samples of deceptive text, on the other hand, are much more difficult to assemble. Genuine deceptive text, by its nature, is made with the intent of fooling someone. This requires the deceiver to construct their deception in a manner that makes it look convincing. It is more difficult to source deceptive text samples in the wild due to the average human’s poor ability to identify deceptive text (Levine & Bond, 2014; Ott, Cardie, & Hancock, 2013). It is possible to source deceptive samples in a traditional method of soliciting entries from subjects in exchange for compensation, such as with the Ott Deceptive Opinion Spam dataset (Ott, Choi, Cardie, & Hancock, 2011), but this can lack the secondary factor of trying to make the deceptive text actively fool the reader. That is, while it is simple to obtain data that is *false*, there is little incentive on the part of the subjects to make the deception *convincing*, and therefore closer to a real-life sample. As (Fornaciari, Cagnina, Rosso, & Poesio, 2020) observed, crowd-sourced online reviews are generally significantly different from wild examples. It is not necessary, after all, to put effort into making a convincing lie if all that is required of a subject is to produce an arbitrary sample.

To rectify this problem, we propose the Motivated Deception Corpus, with the goal of improving the quality of deceptive text through incentivizing higher-quality deception. The incentive arises from the nature of the data collection, which takes the form of the game Two Truths and a Lie. This game revolves around the idea of presenting fellow players with a selection of stories, one of which is false. The other players must figure out which story is the lie while at the same time creating their own stories to fool other players. The structure of the game means that the player that is the best at making their lies believable and determining the lies of other players is the one most likely to win. By using a competitive structure, the subjects are motivated to make their deception convincing if they want to perform well in the game and thus be well rewarded. Using this technique, we have amassed a large amount of high-quality deceptive text to be used in natural language research. This corpus also reaches beyond the simple text and includes behavioral data as well. Every keystroke that the subjects made was recorded as they wrote the stories, including keystrokes that were later deleted by the subject. The timestamps of the keystrokes in milliseconds are recorded alongside them as well.

Lie detection has been studied and practiced in various forms for years. Perhaps the most widely known method of detecting deception is the polygraph (Council, 2003) or one of its derivatives: a system that examines physiological responses such as increased sweat or heart rate that are expected to occur when people lie. Audio-visual analysis of voices and facial expressions is also popular, and has inspired datasets such as the video-based database of deception gathered by Lloyd et al., (2019). On the more verbal side of the spectrum are the analyses described by (Fitzpatrick, Bachenko, & Fornaciari, 2015), who focus on clusters of features present in verbal—as well as physiological and gestural—behaviors. They too note the difference between data collected in a laboratory and the “real world.” There is also the work done by (Abouelenien, Pérez-Rosas, Mihalcea, & Burzo, 2016), which incorporates physiological, linguistic, and thermal recordings to create a more accurate deception-detection system. For the most part, these approaches are ineffective in text, where there are no physiological clues (Ott et al., 2011), but they establish how it is possible to use data of multiple types to improve detection accuracy.

One example of deception from everyday life is in false reviews, or Deceptive Opinion Spam. Reviews make up a truly massive amount of text data, with the Amazon Customer Reviews Dataset alone comprising over 100 million reviews (Amazon, 2014). Problems arise, however, when these reviews are not truthful. This usually takes the form of a malicious customer posting fake negative reviews to hurt a business, or a company shill posting fake positive reviews to inflate its image. Fornaciari and Poesio (2014) observed this type of review in the plethora of these so-called “sock-puppet” reviews of books that were in truth written by the book’s author to drum up sales. They also note the difficulty in labeling ‘real’ deceptive samples, and how they were forced to identify cues that they believed indicated deception without being able to know the absolute ground truth. This is a common problem because humans are ineffective at detecting deceptive text, faring little better than chance (Vrij, 2008; Levine & Bond, 2014; Ott et al., 2013). Humans, in fact are extremely poor even at identifying if a review is generated by a human or artificial intelligence (Hovy, 2016). This is in stark contrast to other linguistic tasks such as sentiment analysis (e.g., identifying if a text sample is praising or condemning something) where humans perform extremely well (Vogler & Pearl, 2019). Part of the purpose of this corpus is to provide an opportunity to evaluate human performance on deceptive text in an environment where they will be motivated to perform well.

Another example of widespread influential deceptive text that has risen to prominence is fake news. This usually takes the form of misinformation or disinformation presented as fact, either on a traditional news source or a social media source such as Facebook. While not a new phenomenon, the subject of fake news has come under increased scrutiny in recent years (Kalsnes, 2018). Fake news, misinformation, or even simply the fear of it can influence people’s perceptions of current events, which can influence their political and social views. It is especially prevalent on social media, where the nature of the medium, such as the short time between event and reporting as well as the diversity of the subject matter, makes detecting falsity a singularly difficult challenge (Shu, Sliva, Wang, Tang, & Liu, 2017). Some datasets, like the one developed by (Tasnim, Hossain, & Mazumder, 2020), try to combat this problem by including contextual information and spatiotemporal data. These false stories impact consumers directly by influencing decisions made that affect the reader’s own life. For example, populations that are subject to misinformation about crucial vaccines such as the COVID-19 vaccine become less likely to inoculate themselves and their dependents, which can have far-reaching effects (Carrieri, Madio, & Principe, 2019).

Deceptions like fake news, while often expressed through the medium of text, are actually difficult to compare to occurrences like Deceptive Opinion Spam. The intent and purpose behind them, other than simply obscuring the truth, is usually wildly different. Fake news tells stories in a way that fake reviews do not. While some reviews can certainly contain a narrative, it is not essential to have one to qualify as a review. Fake news cannot operate this way beyond the basic reporting (or misreporting) of facts; a narrative is essential to qualify as news. Our corpus is more similar to fake news or fake forum posts than false reviews, as both involve primarily a type of storytelling.

### Machine learning efforts

Compared to classical lie detection, the amount of data for text-based deception is relatively small. Ott et al., (2011) developed the Ott Deceptive Opinion Spam corpus, which consists of 800 true reviews from TripAdvisor and 800 deceptive reviews sourced from Amazon Mechanical Turk. The Ott corpus is one of the most commonly used gold-standard corpora in deception detection tasks. However, it suffers from the fact that the Mechanical Turkers that created the deceptive samples were only asked and compensated for arbitrary samples; there was no additional incentive to be convincing. It is also, despite being of considerable size for a deceptive corpus, relatively small compared to corpora of other types. The Amazon Customer Reviews Dataset, by comparison, contains an enormous amount of data, on the order of 100 million samples, but while some are undoubtedly false, there is no easy way to identify them as there is no deceptive label (Amazon, 2014). There is also the DeRev dataset made by Fornaciari and Poesio (2014), which identifies a number of helpful clues in detecting deceptive reviews, but still ultimately relies on human experts to identify said deception. Wang (2017) created the LIAR dataset based on fake news as determined by Politifact. This dataset is high quality, as long as Politifact is reliable, but is difficult to scale, since it requires manual fact-checking of each individual story, and prone to subjective interpretations of p`olitical text. Other efforts in fake news, such as in Aphiwongsophon and Chongstitvatana (2018), use a variety of techniques including support vector machines, naive Bayes algorithms, and neural networks to separate fake news from real. (Feng, Banerjee, & Choi, 2012) assembled a dataset of 800 true and false reviews identified by Yelp’s filter system, however Yelp’s criteria for identification are not publicly disclosed.

There are a few corpora that while not true deceptive text corpora are similar enough in premise to make them worthwhile to explore when examining deceptive text. Filatova (2012) created a corpus of Amazon reviews that were labeled as ironic (or sarcastic) or normal. These reviews were gathered and labeled by Amazon Mechanical Turkers and number 1254 samples in total. While these samples share qualities with deceptive text in that both are not presenting the unvarnished truth, it is difficult to use this corpus as a deceptive corpus. The labeling is crowdsourced from non-experts and can be highly subjective. Furthermore, irony cannot safely be put in the same category as true deception as while neither is technically telling the truth, irony and deception have inherently different goals of intention. Irony is meant to be noticed, difficult though that is in a text setting, while deception is not. The Self-Annotated Reddit Corpus (SARC) of sarcasm by (Khodak, Saunshi, & Vodrahalli, 2017) is made of 1.3 million Reddit comments, which are labeled by the commenter. The size of this corpus is impressive, although it relies on the Reddit user submitting the comment to self-report the sarcasm. In this corpus, a comment ending in a ‘/s’ is flagged as sarcastic and one without that ending is genuine. This allows for mass scraping of Reddit comments, but this nomenclature is not universally followed. Like Filatova et al.’s corpus, it also cannot be directly used as a deceptive corpus due to the inherent differences between true deception and sarcasm, although some techniques can be applied to both types of text.

Previous attempts to perform deception detection often rely on techniques such as support vector machines and linguistic characteristics. One of the earliest is the work done by (Newman, Pennebaker, Berry, & Richards, 2003), which investigated linguistic clues to determine differences between truthful and deceptive statements. Vogler and Pearl (2019) used a support vector machine operating on linguistically defined features to classify the Ott corpus. They were able to achieve an accuracy of 87% using this method. Xu and Zhao (2012) train a maximum entropy model on the Ott corpus and were able to achieve 91.6% accuracy. (Li, Ott, Cardie, & Hovy, 2014) tried to find a general rule for identifying deceptive opinion spam using features like part-of-speech on several datasets including the Ott corpus, achieving 81.8% accuracy on Ott. Ren and Ji (2017) expand on this work by using a recurrent neural network on the same data, improving the accuracy to 85.7%.

On the neural network side, several interesting tools have arisen to process textual input. Hu (2019) used a variety of models to identify concealed information in text and verbal speech, best among them a deep learning model based off bidirectional LSTMs. Concealed information, in this context, refers to when a person has knowledge about a subject and is withholding it, as compared to Hu’s definition of deception where someone fakes knowledge they do not have. Hu created a corpus of wine tasters evaluating wines and encoding in various ways such as n-grams, LIWC, and GloVe embeddings (Pennington, Socher, & Manning, 2014) based on the recordings. The LSTM model using these features achieved an f-score in identifying the presence of concealed information of 71.51, defeating the human performance of 56.28.

One of the standout neural network models in working with text-based tasks is the Bidirectional Encoder Representations from Transformers (BERT). The groundwork for this model was laid by Vaswani et al., (2017), who developed a new kind of network based on self-attention that showed dramatic improvements in the area of machine translation. Devlin et al. built on this work to create the structure known as BERT, using many instances of self-attention networks to learn contextual representations of text. BERT has proven highly competitive in multiple areas including sentiment classification and next sentence prediction (Devlin, Chang, Lee, & Toutanova, 2018). (Barsever, Singh, & Neftci, 2020) were able to utilize BERT in order to perform deception detection on the Ott dataset, proving its viability and power in that arena and setting the state of the art. Their model was able to achieve an accuracy of 93.6% on the Ott Deceptive Opinion Spam corpus. They also used BERT as a generative model to produce machine-created samples of both truth and deception, and identified some linguistics trends such as deceptive samples being less varied in the parts of speech that they used.

### Our corpus

With this Motivated Deception corpus, we introduce a set of deceptive text that is large, realistic, and challenging. This will provide new avenues and benchmarks to researchers working in the field of deceptive text. It includes both raw text and behavioral data in the form of the keystroke timestamps, combining textual data and behavioral data.

In addition to the corpus itself, we provide several machine learning benchmarks on the accuracy and hit rate of the text in several configurations. These should form useful guidelines for researchers aiming to evaluate the efficacy of their own methods on this corpus. None of these experiments were formally preregistered. The data collected is available https://github.com/danbarsever/Motivated-Deception-Corpus.

## Methods

Data were sourced from 177 University of California, Irvine undergraduate students between 18 and 29 years of age using the Experimental Social Science Laboratory Sona system at UCI. Seventeen subjects were determined to be operating in bad faith (submitting mutually exclusive ‘true’ stories and other suspicious behavior) and were removed as bad actors, for a total of 160 subjects. The game was developed in oTree Studio (Chen, Schonger, & Wickens, 2016) and deployed on a Heroku server in order to make it accessible from any computer with a link to the study. All subjects were paid a $7.00 show-up fee as well as additional rewards based on how they performed in the game. Sessions were online and consisted of between 20 and 50 subjects participating synchronously at their computers with a mouse and keyboard. All instructions were text-based. The time remaining for each page was clearly visible on all pages. All data collection was done remotely with a researcher monitoring each session but otherwise not interacting with the subjects. If a subject needed to leave early, the researcher would take over their position in the game and the subject would be compensated for the work they did up to that point. Any data entered by the researcher, as well as entries that were blank or obviously violated the spirit of the game (such as one-word entries and “true” stories that were mutually exclusive) were discarded.

### Two Truths and a Lie

**Fig. 1:**
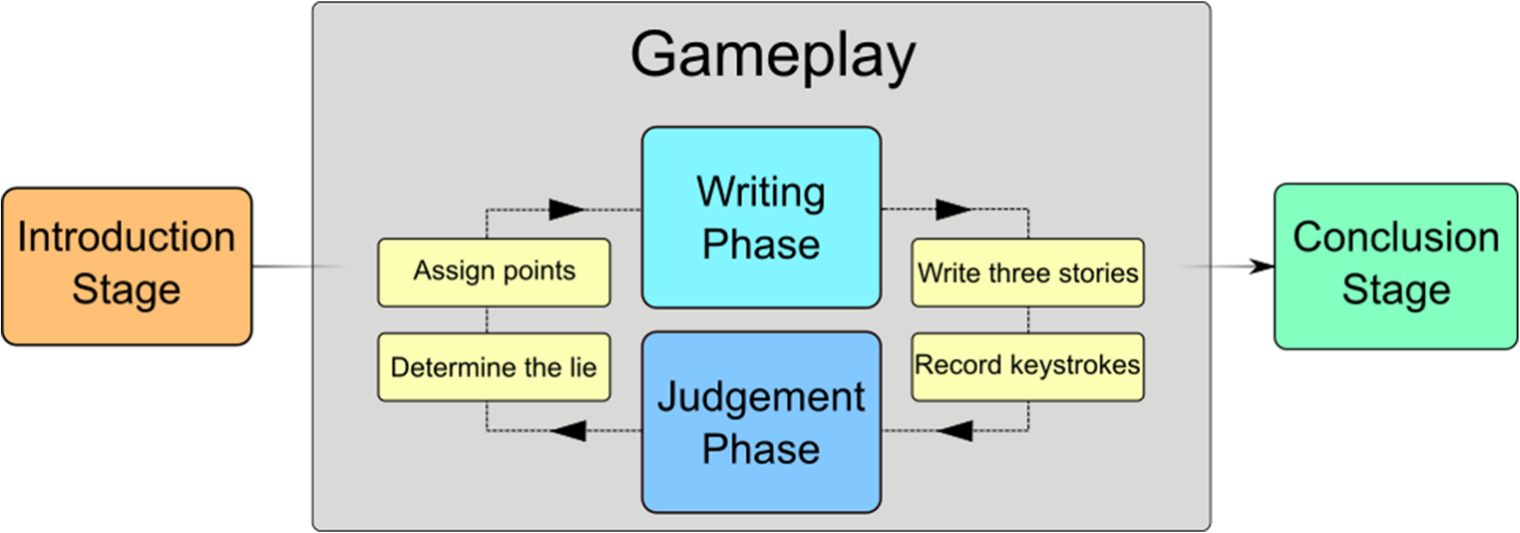
General flow of the Two Truths and a Lie game. First is the introduction phase where the mechanics are explained, followed by the main gameplay loop where stories are written, recorded, and judged. Finally, the scores are tallied in the conclusion and compensation is given to the player

The technique used to gather data for this corpus is based on the game Two Truths and a Lie. The game consisted of an introduction stage, ten rounds of gameplay, and a conclusion stage. At the end of the game, subjects received points based on how well they performed during the game and are compensated proportionally. A general outline of the game can be seen in Fig. 1.

#### The introduction stage

In this phase, subjects are given written instructions on how to play the game, as well as an explanation of the reward system. Subjects could indicate their readiness to move on to the next phase by pressing a button at the bottom of the page, or they would be automatically advanced after 5 min.

#### Gameplay

Subjects participated in ten rounds of gameplay corresponding to ten provided topics (“Homework”, “Vacation”, “Dinner”, “Exams”, “Housing”, “Dating”, “Shopping”, “Family”, “Friends”, and “Fitness”). Each round of gameplay consisted of two phases, a writing phase and a judgement phase.

In the writing phase, subjects were presented with the round’s topic and a text box within which to write a story between 30 and 200 words long. When finished writing the story, they would press a button and move on to another page with a new text box. In this manner, subjects wrote true stories on the first two pages and a lie on the third. Each page would automatically advance after 2 min, and any data entered would automatically be submitted. During this phase, the subject’s keystrokes would be recorded, as well as the timestamp of the keystroke in milliseconds after the page was loaded. Subjects were not informed about this aspect of the data collection. After writing the false story, gameplay would proceed to the judgement phase.

In the judgement phase, each subject’s trio of stories would have its order randomized and sent off to another random subject, so that each subject is viewing one other subject’s story trio. All stories in the trio were presented at the same time. The order of the trio was also shuffled so that the receiving player cannot simply use the position in the list to make the determination. The subject was given 2 min to read the stories and select via button press which one they believed to be the lie. This equates to a three-alternative forced-choice task where the signal being determined is the lie. If no selection was made in time, the page advanced automatically and no choice was recorded. If a subject correctly guessed which of the trio was the lie, they received a point. If a subject guessed incorrectly or did not make a choice, the subject who wrote the trio received a point instead. After all judgements are recorded, gameplay proceeds either to the next round or the conclusion phase if all rounds are completed.

#### Conclusion and payment

In this stage, each subject’s points were tallied up and the subject was told how many points they earned. Subjects could earn between 0 and 2 points per round, totaling to between 0 and 20 points over ten rounds. Each point equated to an additional dollar of reward money. Each subject received a personal code to be inputted in a compensation form as well as a link to post-game survey where they could give feedback on the experiment. Subjects were primarily paid through Venmo, PayPal, and Zelle.

## Results

### Corpus

After playing Two Truths and a Lie with 177 subjects, we have assembled a corpus of deceptive text that is both large and realistic. The linguistic makeup of this corpus is outlined in Tables 1 and 2. The average parts of speech per story (shown in Table 2) were defined and tagged with the Natural Language ToolKit (Loper & Bird, 2002) after each story was tokenized into words. In general, lies tend to be slightly longer with more variance in the length, although the variance in both categories makes it difficult to classify based on that alone. Note the example in Table 3, in this case the lie is significantly longer than either true story, and it was not identified as such. By contrast, Table 4 shows a lie that is of similar or slightly less length than the true stories, but it was correctly identified. This indicates that subjects do not automatically identify longer stories as deceptive. Both categories also tend to be similar in terms of the part-of-speech makeup, as shown in Table 2. The biggest difference is in the number of interjections, where true stories have almost twice as many occurrences.

**Table 1:** Descriptive statistics of the stories generated

Statistic	Value
Average Length Truth (words)	30.08
Standard Deviation Length Truth (words)	17.74
Average Length Lie (words)	31.29
Standard Deviation Length Lie (words)	19.05

**Table 2:** The prevalence of given parts of speech in true and false stories

	Average percentage of story	
Part of Speech	Truth	Lie
Coordinating Conjunction	3.275	3.279
Cardinal Digit	1.489	1.259
Determiner	6.827	7.079
Preposition	10.449	10.483
Adjective	6.120	6.216
Modal	0.728	0.741
Noun	18.833	18.849
Predeterminer	0.0877	0.0894
Possessive Ending	0.154	0.163
Possessive Pronoun	3.379	3.669
Adverb	6.574	6.449
Particle	0.785	0.767
Infinite Marker (to)	2.979	3.177
Interjection	0.0106	0.00610
Verb	18.321	18.415
wh-Determiner	0.209	0.215
wh-Pronoun	0.187	0.183
wh-Adverb	0.735	00.671

All forms of adjectives, adverbs, verbs, and nouns are grouped together

**Table 3:** Example triplet submitted by the subjects

Label	Story
Lie	I have two cousins from my father’s side. I honestly don’t understand why they hate me. I haven’t spoken to them since I was five.
Truth	I have 12 cousins from my mother’s side of the family. None of which I am close to.
Truth	I am currently quarantined with my mother and sister, which I am really thankful for.

The topic for the round was ‘Family’. The player receiving this triplet failed to correctly identify the lie

**Table 4:** Example triplet submitted by the subjects

Label	Story
Lie	I recently went to my relative’s wedding in Philadelphia. It was a fun experience overall. While I was there I was able to go see the Liberty Bell, which was cool.
Truth	I have a large family. I have three younger siblings and I’m the eldest sibling. Because of this, I have added responsibilities and watch my siblings when my parents aren’t home.
Truth	All my grandparents but one died before I was born. I don’t know my living grandparent too well because he lives in another country and doesn’t speak English.

The topic for the round was ‘Family’. The player receiving this triplet correctly identified the lie

After discarding unusable data, we acquired 1572 deceptive samples and 3144 truthful samples along with their corresponding keystroke data. The truthful stories had a mean length of 30.17 words with a standard deviation of 17.95 words. The deceptive stories had a mean length of 31.25 words with a standard deviation of 19.03 words.

To establish whether our corpus truly resembles natural deceptive text, we compared it to an existing corpus, the LIAR dataset (Wang, 2017). The LIAR corpus is composed of statements by politicians and other public figures that have been fact-checked and labeled according to their truthfulness. While it is not cost-efficient to create a large corpus with this method, it does allow the LIAR set to be a solid stand-in for natural ‘wild’ deception. For simplicity, we will be using the ‘true’ and ‘false’ labels of the LIAR dataset and sampling until class sizes are equal.

Our first comparison was through latent semantic analysis, or LSA. To perform LSA, we first converted each document to a term frequency-inverse document frequency (Tf-Idf) vector using the Scikit-learn TfIdf vectorizer (Pedregosa et al., 2011). We then performed singular value decomposition on the vectors to render them into operable matrices, with each row representing a document in the corpus and each column representing a decomposed component. For this comparison, we decomposed each corpus into 100 components. We then measured the similarity of the matrices by taking the RV coefficients of the LIAR corpus to both of the competing datasets (Robert & Escoufier, 1976). An RV coefficient measures the closeness of two matrices using values from -1 to 1, with 1 being an identical matrix. The RV coefficient between the LIAR corpus and the game corpus was 0.995, while the coefficient between LIAR and Ott was 0.799. This is the first indicator that our corpus resembles natural data better than crowdsourced data. Scatter plots of a two-component decomposition can be viewed in the Appendix.

The second comparison is through evaluating the competing performances of networks trained on synthetic data and evaluated on natural data. We take identical instances of our BERT-based network and train one on the Ott Deceptive Opinion Spam corpus, as the most prominent example of crowd-sourced data, and one on our corpus of game-generated data. Training lasted ten epochs. We then test both networks on the test set of the LIAR dataset, without ever training on the LIAR set. Whichever network performs higher will indicate which set of training data more closely resembles the test data.

The Ott-trained network achieved an accuracy of 47.2% on the LIAR corpus, while the game-trained network achieved 53.6% accuracy, a difference of 6.4 percentage points. While the network trained on the game corpus has only a small increase in accuracy from randomness, being trained on the crowdsourced data seems to actively hurt the network’s ability to classify natural samples. Taken together, these two comparisons show that our game-generated Motivated Deception Corpus is a better approximation of natural data than the crowdsourced Ott dataset.

### Human performance

Two Truths and a Lie was not only about recording samples of text, but observing the ability of the human players to judge each others’ samples. In this regard, they were generally ineffective. The average accuracy of the players when judging samples was 35.7%, close to random chance (33%). Using a 95% confidence interval of proportions, we calculated the true mean accuracy of the human population to be between 26.8% and 42.8%. This leaves us unable to conclude that humans are, on average, better than random chance at identifying deception.

**Fig. 2:**
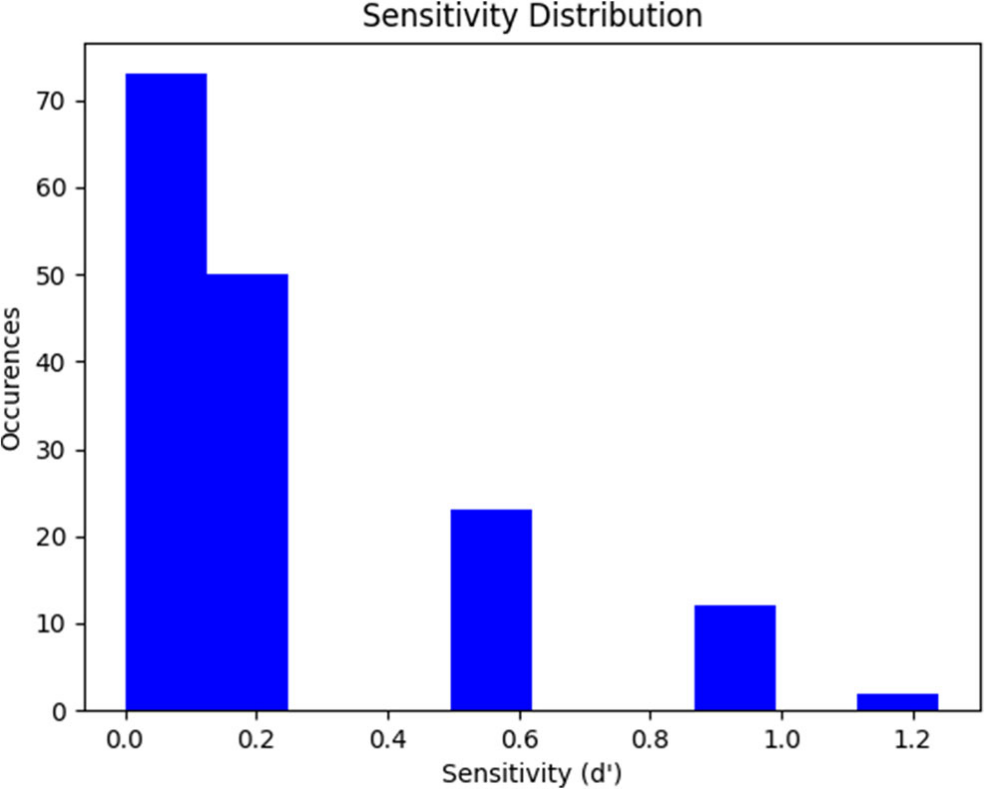
Histogram showing the distribution of sensitivities across the subject pool. Players that scored at chance or below were given a sensitivity score of 0

We also calculated the sensitivity (d’) of each subject. The sensitivity of the subject is a measure of how well they can identify deception when it is present. The sensitivities were derived from the table outlined in (Frijters, Kooistra, & Vereijken, 1980) using the metric for a three-alternative forced-choice task. In total, 45.6% of players were assigned a sensitivity of 0, meaning that they performed at the level of random chance or worse. Only 23.1% managed a sensitivity score better than 0.5. This reinforces the conclusion that the ability of humans to detect truth from lies is weak. A histogram showing the full distribution of sensitivities is shown in Fig. 2.

### Machine learning benchmarks

**Fig. 3:**
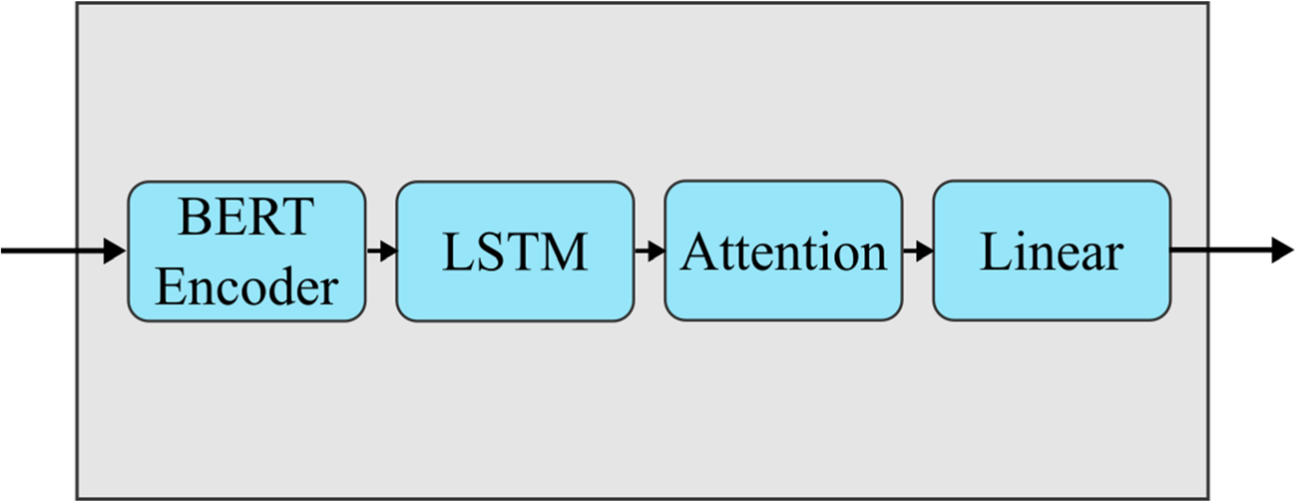
Structure of the discriminator used to classify the corpus data. The standard BERT model is followed by a bidirectional LSTM, a self-attention layer, and a fully connected linear layer

We applied neural networks on the corpus to establish some machine learning benchmarks and compare them to benchmarks on other corpora. A summary of the results can be seen in Table 5. The base classifier we used was the one utilized in Barsever et al., (2020). The structure of this classifier is shown in Fig. 3. The first layer of the classifier is the pre-trained BERT model from the huggingface transformer library (Wolf et al., 2020). The pooled output is extracted from the BERT model and fed into a bidirectional recurrent LSTM layer. The output from this recurrent layer is then fed into a self-attention layer based on the machine translator networks from Vaswani et al., (2017). Finally, the attention layer output is passed through a fully connected linear layer that classifies the input into two possible classes: truthful or deceptive.

**Table 5:** Performance statistics of the BERT classifier

BERT Model	Accuracy	Hit Rate	False Positive	Sensitivity (d’)
3-AFC	41.2%	N/A	N/A	0.260
Binary Classification	57.8%	40.5%	33.5%	0.188
NSP* (Unshuffled)	79.9%	77.4%	17.3%	1.694
NSP (Shuffled)	56.0%	67.2%	55.03%	0.319

^*^ Next sentence prediction

We chose BERT as the basis of our network because of its power and ease of use. BERT, or Bidirectional Encoder Representations from Transformers, performs well in a wide variety of contextually sensitive language tasks due to being able to detect when the meaning of a sequence has changed depending on context. This allows it to detect subtle differences in phrasing (Devlin et al., 2018). It also requires very little preprocessing of the data, allowing samples to be fed directly into the model without adding additional steps or complexity. The modified BERT classifier in Fig. 3, which we use as our default classifier, is able to achieve a 93.6% accuracy on the Ott Deceptive Opinion Spam dataset, making it the state of the art in the field of deception detection. By comparing its results on the Ott dataset with the results achieved on the corpus we made, we can gain a rough idea of how much more challenging our corpus is to classify.

#### BERT configuration 1: Three-alternative forced choice

We tested several configurations of the BERT classifier on the corpus. The configuration we refer to as BERT for Three-alternative Forced Choice, or 3-AFC BERT, is the most directly analogous to the human task. In this configuration, each story in a particular triplet is presented to the classifier in turn, and the strength of the ‘lie’ class neuron is recorded for each one. The story that generated the strongest ‘lie’ reaction is considered the model’s ‘choice,’ allowing the network to ‘pick’ from the three stories in a simulacrum of the humans’ 3-AFC task from the game. This configuration was able to reach an accuracy of 41.2%, outperforming the human subjects, but not significantly. Due to the nature of a 3-AFC task, we cannot calculate a rate for hits or false positives, but using the table from Frijters et al., (1980), we find the sensitivity to be 0.26.

#### BERT configuration 2: Binary classification

The next configuration is a more traditional machine classifier, with simple binary classification. In this mode, BERT is presented with each story individually and attempts to make a true/false classification without any additional context. This configuration was able to achieve an accuracy of 57.8%, however since the number of samples in each class is unbalanced (there are two truths for every lie), it can be difficult to evaluate the network using only accuracy. When looking at the hit rate and false-positive rate, we find that the network managed 40.5% and 33.5% respectively, with a sensitivity score of 0.188. This indicates that the binary configuration is less sensitive than the 3-AFC setup.

#### BERT configuration 3: Next sentence prediction

We also utilized another form of BERT, BERT for Next Sentence Prediction, to perform a slightly modified classification task. This model differs from the base BERT model in both the style of its inputs and the meaning of its output. This model takes two sequences as inputs. The first of these is the *context*, or the ‘first sentence’. The second sequence is referred to as the *query*, or the ‘next sentence.’ The task of the model is to predict whether the query was the next sentence following the context in the original document. At its core, this is still a classification problem, with one class being ‘yes this is the next sentence’ and the other class being ‘this sentence is unrelated.’ We used this basic structure to simulate, to a limited extent, the network playing the game. We first divided the corpus into each triplet of stories submitted by the players. The stories are then assembled into pairs. Each pair consists of one of the true stories as the context and either the remaining truth or the lie as the query. With two truths available to act as contexts and each having two possible queries, this creates four paired samples for each triplet. This has the side effect of balancing the size of each class, as there now an even number of cases where the lie is the query. The network then runs its next-sentence-prediction classification, training on whether or not the query is a lie given its context.

We ran this configuration on two versions of the dataset, which we refer to as shuffled and unshuffled. In the unshuffled version, the query and context are restricted to being from the same triplet. In the shuffled condition, the query and context are selected from separate, randomly selected triplets. We fine-tuned this model on both the shuffled and unshuffled versions of this corpus of paired samples for 100 epochs.

Under the unshuffled condition, we achieved an accuracy of 79.9%, a hit rate of 77.4%, and a false-positive rate of 17.4%. This is the highest performing configuration so far, indicating that BERT can learn contextual clues for a given triplet. However, when the stories are shuffled, all of these measure worsen, particularly the accuracy and false-positive rate, which change to 56% and 55%, respectively. The dramatic drop in performance indicates that BERT cannot generalize cues across different subjects.

## Discussion and future work

Detecting deceptive text is a difficult task, made even more onerous by the lack of comparability between crowdsourced data and real-world data. Between fake news, misleading forum posts, and sock-puppet online reviews, the opportunities for deception only continue to grow. With the ability of online deceptive text to propagate and influence real-world decisions of readers, it is important to have a corpus of data that accurately reflects the level of effort of what a reader is likely to find online. We created a Motivated Deception corpus of text samples that is designed to be closer to real-life deception than other corpora. We did this by incentivizing players of Two Truths and a Lie to both deceive and perceive deception on their fellow players by paying them based on their performance. By turning the collection process into a game, it allows the data collected to be more indicative of real-world samples of deceptive text like fake news and false social media posts. This has created a corpus that is large, realistic, and challenging for both machine classifiers and humans.

The storytelling involved in the game necessitates a caveat when examining this corpus. When creating this corpus, which stories were true and which were lies was self-reported by the participants. While we eliminated several obvious bad actors, it is impossible to verify every subject’s story as genuinely truthful or deceptive. The subjects were aware that the stories could be vetted and their credit could be revoked, but it is still possible that some could have attempted to cheat the system. This is a problem endemic to data collection of this kind, however even with this caveat we believe this corpus is a valuable resource for those looking for motivated samples of deception. The oversight of a researcher during the live data collection and interactions with the subjects indicate that the vast majority operated in good faith and gave genuine entries while playing the game. In general, online subjects tend to be honest when self-reporting (Paolacci and Chandler, 2014), and with few exceptions there has been no reason to assume any different from the players of this game.

The first and most relevant benchmark to assess is how accurately do humans perform the task of identifying deception when they see it. We found that overall, the human subjects performed at around the level of random chance, with almost half having a sensitivity score of zero. This is consistent with the goal of incentivizing the players to produce convincing deception, and thus making the task more challenging, as well as the natural poor ability of humans to recognize deceptive text.

We used the state-of-the-art BERT classifier to generate machine learning benchmarks on this corpus in order to help quantify the level of difficulty compared to previous corpora. We designed several configurations of BERT to provide benchmarks in a variety of contexts. One such configuration was the 3-AFC configuration, presenting each story belonging to a given trio to BERT and recording the output of the ‘lie’ classification neuron, simulating the Three-Alternative Forced-Choice the human subjects faced. This configuration resulted in an accuracy of 41.2%.

In a binary classification task, BERT achieved an accuracy of 57.8%, a hit rate of 40.5%, and a false-positive rate of 33.5% on this corpus. This is an improvement over the human performance. It does, however, indicate that the network has a preference for truth, given that the miss rate is significantly higher than the false-positive rate. This is perhaps to be expected, given that the number of truthful samples is double that of the deceptive samples. When the data was sampled to have equal quantities in both classes we achieved an accuracy of 53.9%, compared to the 93.6% accuracy it achieved on the Deceptive Opinion Spam dataset, marking it as significantly more difficult to classify. When classifying the keystrokes or their timestamps, the accuracy is no better than chance.

Another configuration used the BertForNextSentencePrediction model from huggingface’s transformer library. When using one truthful statement as the context and one other statement from the same trio as the query, the model was able to predict whether the query was truthful or deceptive 76% of the time. This accuracy only exists when predicting on statements from the same triplet however; when the context and query are shuffled so that they are from different triplets the accuracy drops closer to chance (56%).

All the configurations of BERT struggled with this corpus, much more so than when it was used to evaluate the Ott Deceptive Opinion Spam dataset. This shows that our corpus is significantly more challenging than previous corpora. It is our intent that the benchmarks we establish here not be taken as endorsements of our model, but starting lines so that whoever uses this corpus will have a baseline with which to compare their results. We aim for this corpus to drive the creation of more sensitive, nuanced models that can capture the intricacies of realistic deception.

In the future, we plan to further utilize this corpus to collect mass judgements on the stories from a crowdsourcing service such as Amazon Mechanical Turk. This will allow us to identify stories that consistently register as false or true, regardless of their actual level of truth. We plan to use this data to extract what patterns of text signal to humans whether it is deceptive or truthful, rather than what patterns humans put in when they create a sample. We also plan to incorporate the keystroke data we gathered into a model that can use it to inform its decisions beyond what only the text shows.

## Figures and Tables

**Fig. 4:**
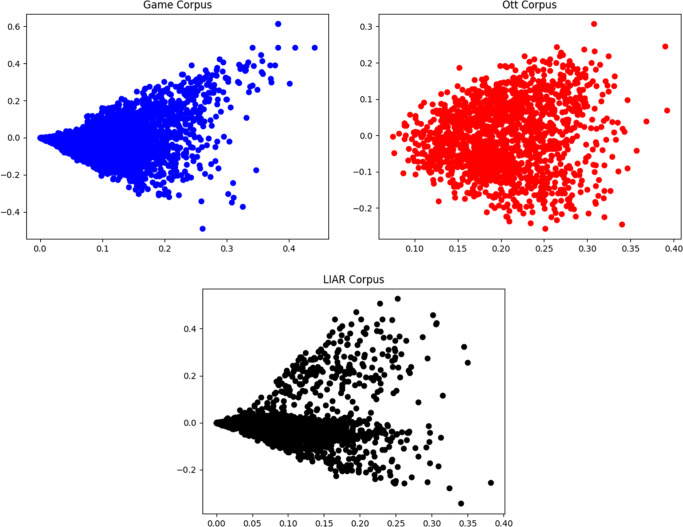
Scatter plots of the two-component singular value decomposition of each corpus after being vectorized with Tf-Idf. The similarity between the shapes of the Game corpus and the LIAR corpus is apparent, as is the dissimilarity between the Ott corpus and the others

## References

[CR1] Abouelenien M, Pérez-Rosas V, Mihalcea R, Burzo M (2016). Detecting deceptive behavior via integration of discriminative features from multiple modalities. IEEE Transactions on Information Forensics and Security.

[CR2] Amazon, A. (2014). Amazon customer reviews dataset. https://s3.amazonaws.com/amazon-reviews-pds/readme.html Aphiwongsophon,S.,&Chongstitvatana,P.(2018)Detectingfakenewswithmachinelearningmethod,528–531. Barsever,D.,Singh,S.,&Neftci,E.(2020)BuildingaBetterLieDetectorwithBERT:TheDifference BetweenTruthandLies,1–7.

[CR3] Carrieri V, Madio L, Principe F (2019). Vaccinehesitancyand(fake)news:Quasi-experimentalevidence fromItaly. Healtheconomics.

[CR4] Chen DL, Schonger M, Wickens C (2016). oTree—Anopen-sourceplatformforlaboratory,online, andfieldexperiments. JournalofBehavioralandExperimentalFinance.

[CR5] Council NR (2003). Thepolygraphandliedetection.

[CR6] Devlin,J.,Chang,M.-W.,Lee,K.,&Toutanova,K.(2018).Bert:Pre-trainingofdeepbidirectional transformersforlanguageunderstanding.arXiv:1810.04805.

[CR7] Feng,S.,Banerjee,R.,&Choi,Y.(2012).Syntacticstylometryfordeceptiondetection.In*Proceedings ofthe50thAnnualMeetingoftheAssociationforComputationalLinguistics:ShortPapers-Volume2*(pp. 171–175): AssociationforComputationalLinguistics.

[CR8] Filatova,E.(2012).Ironyandsarcasm:Corpusgenerationandanalysisusingcrowdsourcing.In*Lrec*(pp. 392–398):Citeseer.

[CR9] Fitzpatrick E, Bachenko J, Fornaciari T (2015). Automaticdetectionofverbaldeception. SynthesisLecturesonHumanLanguageTechnologies.

[CR10] Fornaciari T, Cagnina L, Rosso P, Poesio M (2020). Fakeopiniondetection:howsimilarare crowdsourceddatasetstorealdata?. LanguageResourcesandEvaluation.

[CR11] Fornaciari,T.,&Poesio,M.(2014).IdentifyingfakeAmazonreviewsaslearningfromcrowds.In*Proceedings ofthe14thConferenceoftheEuropeanChapteroftheAssociationforComputationalLinguistics*(pp. 279–287): AssociationforComputationalLinguistics.

[CR12] Frijters J, Kooistra A, Vereijken P (1980). Tablesofd’forthetriangularmethodandthe3-AFCsignal detectionprocedure. Perception&Psychophysics.

[CR13] Hovy,D.(2016).Theenemyinyourowncamp:Howwellcanwedetectstatistically-generatedfakereviews–an adversarialstudy.In*Proceedingsofthe54thAnnualMeetingoftheAssociationforComputationalLinguistics(Volume 2:ShortPapers)*(pp. 351–356).

[CR14] Hu,S.(2019).Detectingconcealedinformationintextandspeech.In*Proceedingsofthe57thAnnualMeetingof theAssociationforComputationalLinguistics*(pp. 402–412).

[CR15] Kalsnes,B.(2018).Fakenews.In*OxfordResearchEncyclopediaofCommunication*.

[CR16] Khodak,M.,Saunshi,N.,&Vodrahalli,K.(2017).Alargeself-annotatedcorpusforsarcasm. arXiv:1704.05579.

[CR17] Levine TR, Bond CF (2014). Directandindirectmeasuresofliedetectiontellthesamestory:Areply totenBrinke,Stimson,andCarney(2014). PsychologicalScience.

[CR18] Li,J.,Ott,M.,Cardie,C.,&Hovy,E.(2014).Towardsageneralruleforidentifyingdeceptiveopinionspam. In*Proceedingsofthe52ndannualmeetingoftheassociationforcomputationallinguistics(Volume1:LongPapers)*,(Vol. 1pp. 1566–1576).

[CR19] Lloyd EP, Deska JC, Hugenberg K, McConnell AR, Humphrey BT, Kunstman JW (2019). MiamiUniversitydeceptiondetectiondatabase. BehaviorResearchMethods.

[CR20] Loper,E.,&Bird,S.(2002).NLTK:thenaturallanguagetoolkit.arXiv:cs/0205028.

[CR21] Newman ML, Pennebaker JW, Berry DS, Richards JM (2003). Lyingwords:Predicting deceptionfromlinguisticstyles. PersonalityandSocialPsychologyBulletin.

[CR22] Ott,M.,Cardie,C.,&Hancock,J.T.(2013).Negativedeceptiveopinionspam.In*Proceedingsofthe 2013conferenceoftheNorthAmericanChapterOfTheAssociationForComputationalLinguistics:humanlanguage technologies*(pp. 497–501).

[CR23] Ott,M.,Choi,Y.,Cardie,C.,&Hancock,J.T.(2011).Findingdeceptiveopinionspambyanystretch oftheimagination.In*Proceedingsofthe49thannualmeetingoftheassociationforcomputationallinguistics:Human languagetechnologies-volume1*(pp. 309–319):AssociationforComputationalLinguistics.

[CR24] Paolacci G, Chandler J (2014). InsidetheTurk:UnderstandingMechanicalTurkasaparticipantpool. CurrentDirectionsinPsychologicalScience.

[CR25] Pedregosa F, Varoquaux G, Gramfort A, Michel V, Thirion B, Grisel O, Blondel M, etal. (2011). Scikit-learn:MachinelearninginPython. TheJournalofMachineLearningResearch.

[CR26] Pennington,J.,Socher,R.,&Manning,C.(2014).Glove:Globalvectorsforwordrepresentation.In*Proceedings ofthe2014conferenceonempiricalmethodsinnaturallanguageprocessing(EMNLP)*(pp. 1532–1543).

[CR27] Ren Y, Ji D (2017). Neuralnetworksfordeceptiveopinionspamdetection:Anempiricalstudy. InformationSciences.

[CR28] Robert P, Escoufier Y (1976). Aunifyingtoolforlinearmultivariatestatisticalmethods:theRV-coefficient. JournaloftheRoyalStatisticalSociety:SeriesC(AppliedStatistics).

[CR29] Shu K, Sliva A, Wang S, Tang J, Liu H (2017). Fakenewsdetectiononsocialmedia:Adata miningperspective. ACMSIGKDDExplorationsNewsletter.

[CR30] Tasnim S, Hossain MM, Mazumder H (2020). ImpactofrumorsandmisinformationonCOVID-19 insocialmedia. JournalofPreventiveMedicineandPublicHealth.

[CR31] Vaswani,A.,Shazeer,N.,Parmar,N.,Uszkoreit,J.,Jones,L.,Gomez,A.N., ..., etal. (2017).Attentionisallyouneed.In*Advancesinneuralinformationprocessingsystems*(pp. 5998–6008).

[CR32] Vogler N, Pearl L (2019). Usinglinguistically-definedspecificdetailstodetectdeceptionacrossdomains. NaturalLanguageEngineering.

[CR33] Vrij A (2008). Detectingliesanddeceit:Pitfallsandopportunities.

[CR34] Wang,W.Y.(2017).“liar,liarpantsonfire”:Anewbenchmarkdatasetforfakenewsdetection.arXiv:1705.00648. Wolf,T.,Chaumond,J.,Debut,L.,Sanh,V.,Delangue,C.,Moi,A., ..., etal.(2020) Transformers:State-of-the-artnaturallanguageprocessing,38–45.

[CR35] Xu,Q.,&Zhao,H.(2012).Usingdeeplinguisticfeaturesforfindingdeceptiveopinionspam.Proceedingsof COLING2012:Posters1341–1350.

